# Effects of Video Game Training on Measures of Selective Attention and Working Memory in Older Adults: Results from a Randomized Controlled Trial

**DOI:** 10.3389/fnagi.2017.00354

**Published:** 2017-11-01

**Authors:** Soledad Ballesteros, Julia Mayas, Antonio Prieto, Eloísa Ruiz-Marquez, Pilar Toril, José M. Reales

**Affiliations:** ^1^Studies on Aging and Neurodegenerative Diseases Research Group, Universidad Nacional de Educación a Distancia, Madrid, Spain; ^2^Department of Basic Psychology II, Universidad Nacional de Educación a Distancia, Madrid, Spain; ^3^Department of Methodology of the Behavioral Sciences, Universidad Nacional de Educación a Distancia, Madrid, Spain

**Keywords:** selective attention, cognitive training, healthy aging, video games, working memory, Clinicaltrials. gov ID: NCT02796508, https://clinicaltrials.gov/ct2/show/NCT02796508

## Abstract

Video game training with older adults potentially enhances aspects of cognition that decline with aging and could therefore offer a promising training approach. Although, previous published studies suggest that training can produce transfer, many of them have certain shortcomings. This randomized controlled trial (RCT; Clinicaltrials.gov ID: NCT02796508) tried to overcome some of these limitations by incorporating an active control group and the assessment of motivation and expectations. Seventy-five older volunteers were randomly assigned to the experimental group trained for 16 sessions with non-action video games from *Lumosity*, a commercial platform (http://www.lumosity.com/) or to an active control group trained for the same number of sessions with simulation strategy games. The final sample included 55 older adults (30 in the experimental group and 25 in the active control group). Participants were tested individually before and after training to assess working memory (WM) and selective attention and also reported their perceived improvement, motivation and engagement. The results showed improved performance across the training sessions. The main results were: (1) the experimental group did not show greater improvements in measures of selective attention and working memory than the active control group (the opposite occurred in the oddball task); (2) a marginal training effect was observed for the *N*-back task, but not for the Stroop task while both groups improved in the Corsi Blocks task. Based on these results, one can conclude that training with non-action games provide modest benefits for untrained tasks. The effect is not specific for that kind of training as a similar effect was observed for strategy video games. Groups did not differ in motivation, engagement or expectations.

## Introduction

Aging produces declines in several cognitive processes, especially in executive function and attentional control, mediated by the dorsolateral prefrontal cortex. These brain areas as well as the hippocampus suffer the highest degree of age-related atrophy (Raz et al., 2005). Moreover, the prefrontal cortex facilitates the organization and contextualization of incoming information and interacts with the hippocampus when carrying out working memory (WM) tasks (Baddeley, 2003; Dennis et al., 2008; Spaniol et al., 2009). The failure of these basic cognitive abilities is a significant predictor of older adults' difficulties with the instrumental activities of daily living, leading to loss of independence (Owsley et al., 2002). Therefore, it is vital to investigate whether cognitive decline can be reversed or delayed through cognitive training interventions (Ball et al., 2007).

The efficacy of computer-based cognitive training to improve executive functions, including selective attention and working memory in older adults has been extensively investigated (Lussier et al., 2015; see Ballesteros et al., 2015a, for a review). Executive functions are central to most cognitive processes (Barkley, 2001). Selective attention refers to the ability to focus on the task at hand while simultaneously suppressing (inhibiting) irrelevant or distracting information. This ability is closely related to the quantity of the information stored in working memory. Selective attention filters out irrelevant information, enhancing encoding, and maintenance of information in working memory (Blacker et al., 2014). This is important, as WM is a capacity-limited cognitive system responsible for temporarily storing and actively processing information needed for ongoing cognition. This cognitive system is vital to keep information in mind while performing complex tasks such as, comprehension and reasoning (Baddeley and Hitch, 1974). This key component of cognition declines in healthy aging (Park et al., 2002; Bopp and Verhaeghen, 2005) and more profoundly in patients with Alzheimer's disease (e.g., Baddeley et al., 1991; Belleville et al., 2007; Huntley and Howard, 2010) and type 2 diabetes mellitus (e.g., Redondo et al., 2016; see Monette et al., 2014; Mayeda et al., 2015 for meta-analyses), amongst others. A question of great practical relevance is whether WM training methods are effective in older adults, their effect size, their cost effectiveness, and how they affect untrained tasks, in near (between very similar but not identical contexts) and far transfer (between contexts that appear on the surface to be remote and unrelated to each other).

Results of training studies are mixed. Some studies have shown positive transfer effects in young adults (e.g., Brehmer et al., 2012; Blacker et al., 2014; Maraver et al., 2016) and in older adults (e.g., Buschkuehl et al., 2008; Borella et al., 2010, 2014; Heinzel et al., 2014; Toril et al., 2016) while others have reported negative results (e.g., Dahlin et al., 2008; Zinke et al., 2012; von Bastian et al., 2013; Ballesteros et al., 2014; Bürki et al., 2014; Kable et al., 2017). A meta-analysis of training studies conducted with older adults reported improvements in tasks similar to the trained tasks (near transfer) as well as small far-transfer effects (Karback and Verhaeghen, 2014). However, despite the positive results of some training studies, far transfer effects have been questioned. A recent meta-analysis of working-memory training studies with pre-post design and control groups (87 publications) reported reliable improvements immediately after training on measures of verbal and visuospatial WM, but these specific training effects did not generalize to other cognitive skills (Melby-Lervåg et al., 2016).

### Methodological issues

Researchers are increasingly using new technology, including cognitive training platforms and video games, to investigate its impact on cognition, brain plasticity and aging (e.g., Basak et al., 2008; Mozolic et al., 2011; Buitenweg et al., 2012; Boot et al., 2013; Ballesteros et al., 2014; Anguera and Gazzaley, 2015; Boot, 2015; Binder et al., 2016; Toril et al., 2016). The idea that video games could enhance aspects of older adults' cognition has attracted the interest of researchers and led to a great explosion of software devoted to brain training (Anguera et al., 2013; Baniquet et al., 2013; Ballesteros et al., 2015a). However, a number of methodological concerns have been raised related to the efficacy and validity of video-game training studies (Boot et al., 2011; but see Green et al., 2014). A recent extensive review (Simons et al., 2016) concluded: “practicing a cognitive task consistently improves performance on that task and closely related tasks, but the available evidence that such training generalizes to other tasks, or to real-world performance, is not compelling” (p. 173). Non-specific factors like expectancy, motivation, and engagement, as well as the quality of the active control group, are important aspects of the intervention design that should be taken into account to be certain that computerized cognitive training is a good method for enhancing cognition (Motter et al., 2016).

An effective cognitive intervention in older adults should show a transfer of training gains to untrained tasks. It is also of paramount importance that the intervention encourages compliance. Older adults prefer mentally challenging games (Nap et al., 2009), while studies with young adults have shown that fast-paced action games result in broader transfer effects (Green and Bavelier, 2003; Baniqued et al., 2014; for a review see Bavelier et al., 2012).

A systematic review (Kueider et al., 2012) and several meta-analyses suggest that playing video games improves information processing, with interesting larger effects in old-older adults than in young-older adults (Powers et al., 2013; Lampit et al., 2014; Toril et al., 2014). A recent meta-analysis of action video game training studies found that healthy young and older adults benefited from training in overall and specific cognitive domains, but that young adults benefited more than older adults (Wang et al., 2016). These findings suggest the potential of video-game training as an intervention tool for cognitive improvement.

In a previous RCT study (Ballesteros et al., 2014), two groups of older adults participated in 20 1-h training sessions with non-action games or were assigned to a passive control group. Groups were similar at baseline on demographics, vocabulary, global cognition, and depression status. The results showed improvements in the video-game group and no change in the control group in processing speed, attention, immediate, and delayed visual recognition memory, and a trend to improve in Affection and Assertivity, two dimensions of the Wellbeing Scale (Nieboer et al., 2005). However, visuospatial WM and executive control (shifting strategy) functions did not improve. These enhancements in processing speed, selective attention, and spatial memory disappeared after a 3-month non-contact period (Ballesteros et al., 2015b), suggesting that cognitive plasticity can be induced in healthy older adults by training, but that periodic boosting sessions are needed to maintain the benefits.

In a more recent intervention study, we investigated specifically the effects of video game training on visuospatial WM and episodic memory in healthy older adults after 15 1-h sessions playing six non-action video games. Training produced significant improvements compared to a passive control group in two visuospatial WM tasks (Corsi blocks and Jigsaw puzzle task) and other episodic and short-term memory tasks. Gains in the Jigsaw puzzle task, short-term memory, and episodic memory were maintained over a 3-month follow-up period (Toril et al., 2016). In both studies, we compared the performance of experimental groups trained with non-action video games with that of passive control groups who participated in discussion groups on themes related to aging (Ballesteros et al., 2014, 2015b) or who attended courses in the community center for older adults (Toril et al., 2016). It could be argued that participants in the control group who simply met the trainer several times would not expect to improve as much on the transfer tasks as those who received video-game training (Boot and Kramer, 2014; Melby-Lervåg et al., 2016; Simons et al., 2016). Expectancy can influence training results through the placebo effect. In the present RCT, we address several of these significant issues in cognitive training research (Boot et al., 2011, 2013; see Baniqued et al., 2014; Blacker et al., 2014).

### The current randomized controlled trial

To attribute possible training-related improvements to the intervention and avoid placebo effects (Boot et al., 2011; Foroughi et al., 2016), the current RCT (Ballesteros et al., 2017) compared performance on a series of transfer tasks of an experimental group playing selected adaptive non-action video games from *Lumosity* (http://www.lumosity.com) with that of an active control group. The active control group had the same number of sessions playing *The Sims* (Electronic Arts Inc.), a simulation strategy game in which the player takes control of the life of a character in everyday activities, and *SimCity*, a life simulation game in which the player is the Mayor of a city that he or she must expand. Unlike the non-action games, control games were not adaptive (the difficulty was not adjusted over the training to the actual level of performance of the trainee). Some results suggest that adaptive computerized training regimes may improve executive functioning (e.g., Ball et al., 2002; Dahlin et al., 2008; Morrison and Chain, 2011). Both groups used a mobile tablet device to play. At the end of the assessment session, participants in the current study reported their expectations (increase or decrease) regarding their performance on the assessment tasks, using a 5-point Likert scale. Moreover, at the 1st, 8th, and 16th training sessions, participants responded to questions about motivation and engagement for each of the video games.

In sum, we investigated possible cognitive and neural changes in attention and working memory functions in healthy older adults trained in small groups with adaptive non-action video games selected from *Lumosity* for 16 sessions in the presence of the trainer. Their performance on two attentional and two working-memory tasks was compared pre- and post-training with that of an active control group who played a simulation strategy games for the same number of sessions. The electrophysiological data recorded to assess possible neural changes will not be presented in this paper. The objectives of this study were as follows. First, to examine possible effects of playing adaptive non-action video games on older adults' performance on a series of cognitive tasks designed to assess selective attentional functions, mainly distraction and alertness (Oddball Task), effortful (Stroop) and automatic inhibition (Negative Priming), and working memory, mainly maintenance and updating (N-back task and Corsi blocks) in verbal and visuospatial working memory. Second, to explore whether motivation, engagement, and expectations account for possible training-related improvements. We hypothesized that the non-action, adaptive video game group would show greater improvements in selective attention (exhibiting less distraction, more alertness, and better effortful inhibition after training), and enhanced working memory (maintenance and updating) than the active control group.

## Materials and methods

### Participants

Participants were volunteers recruited from several older adult groups attending lectures and courses for senior citizens in UNED Associated Centers in Madrid. Eligible participants were randomized into the cognitive non-action video-game training group and the active control group in which participants played a simulation game. Exclusion criteria were self-reported neurological, psychiatric, or addictive disorders. All the participants lived independently, with normal or corrected-to-normal hearing and vision and were free of neurological and psychiatric disorders, or traumatic brain injury. To determine their eligibility, each participant completed a screening battery consisting of the Mini-Mental State Examination (MMSE; Folstein et al., 1975) to rule out possible cognitive impairment (cut-off score of 27 out of a maximum of 30 points), the Yesavage Depression Scale (Yesavage et al., 1983) to screen for depression (more than six points), and the Information subtest of the WAIS-III scale (Wechsler, 1999). Exclusion criteria were a diagnosis of dementia, cognitive impairment (score of <27 on the Mini-Mental State Examination, MMSE), <20/60 vision with or without correction, inability to complete the study activities, or communication problems. Demographic data and screening test scores corresponding to each group are summarized in Table 1. *T*-tests showed that groups did not differ on these measures (all *p*s > 0.05) at pre-test.

**Table 1:** Demographic information for participants in each group.

**Characteristics**	**Experimental Control *n* = 30**	**Control Group *n* = 25**	***t*-test**	***p***
Age (Years)	66.40 (5.64)	64.52 (4.51)	1.34	0.31
GDS	2.03 (2.35)	1.16 (1.49)	1.60	0.11
MMSE	28.70 (1.29)	29.00 (0.91)	0.97	0.33
Information (WAIS)	20.80 (3.53)	22.12 (2.90)	−1.39	0.17
Educational level	15.9 (4.55)	17.3 (0.27)	−0.25	0.80

*Mean and Standard deviations (in parentheses). Experimental group age range: 55–84 years; 1 participant 60 years or younger and 2 participants 75 years or older. Active control group age range: 55–76; 3 participants 60 years or younger; 2 participants 75 years or older*.

**Figure 1:**
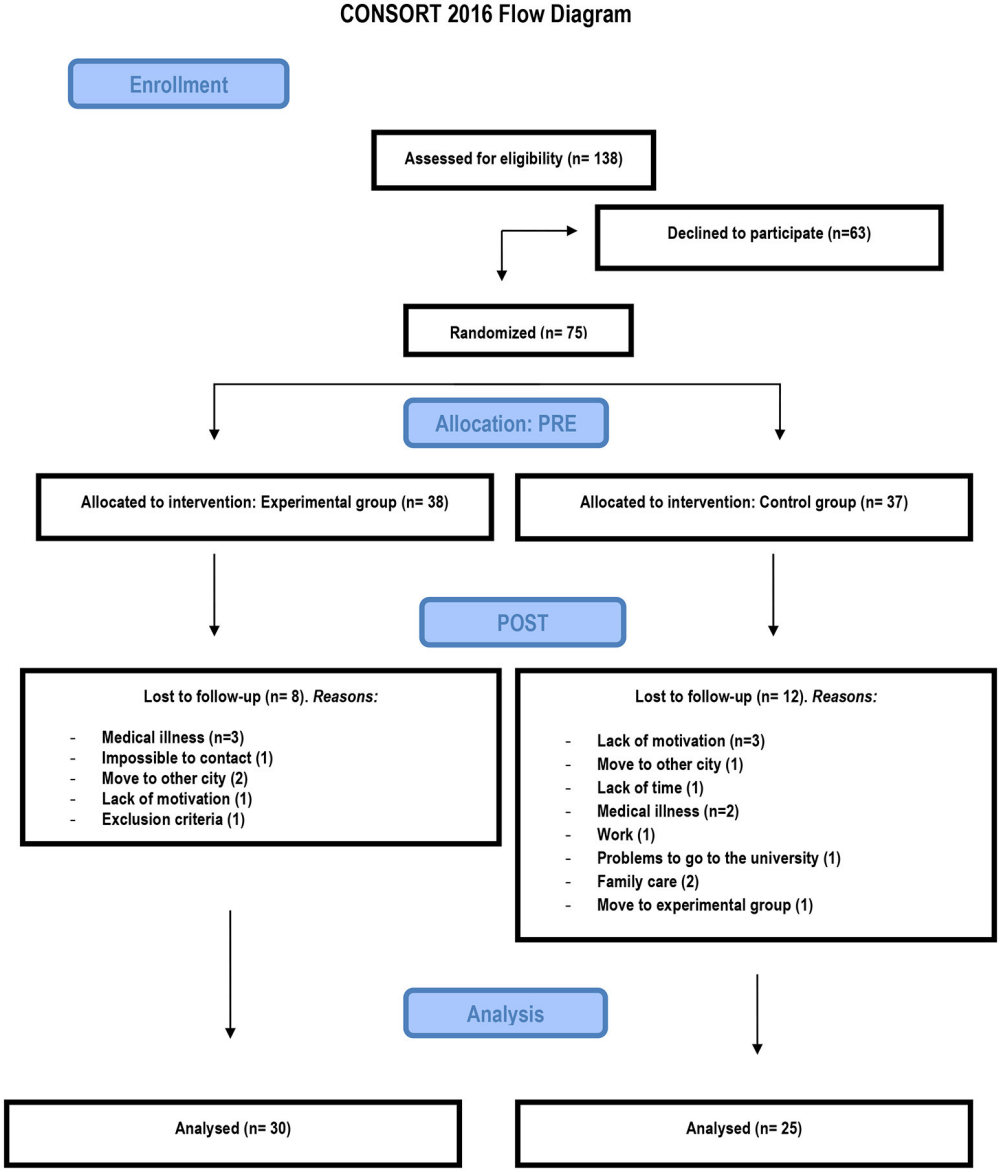
Consort flowchart.

Twenty participants (26.6%) were lost at post-test. The study was completed by 30 of the 38 participants in the non-action video game training group and by 25 of the 37 in the active control group. Analyses of background characteristics showed no differences between dropouts and participants remaining in the respective group. Figure 1 shows the CONSORT Flow diagram of the present study.

Transfer of training was measured as performance improvement at post-test relative to pre-test (baseline) on untrained tasks, measuring selective attention, and working memory. To explore successful transfer of training gains to attentional mechanisms and working memory, data were recorded and analyzed at pre-training (T1) and post-training (T2). Participants in both groups (experimental and active control) completed pre-test and post-test assessments individually in the laboratory. The assessment lasted ~3 1/2 h (including rest periods). It included a Cross-modal oddball task and a Stroop-Negative Priming task to assess the effects of video-game training on attention and top-down control mechanisms, and a *n*-back task and the Corsi Blocks task to assess working memory.

All the methodological designs of the outcome measures were constructed using the rules of counterbalancing and stimulus rotation. Response keys were counterbalanced across conditions. All computerized cognitive tasks were programmed with E-Prime 2.0 (Psychology Software Tools Inc., Pittsburg, PA, USA). The statistical analyses of the behavioral results were conducted with SPSS (version 22). Results were considered significant at *p* < 0.05, with Bonferroni-corrected *post-hoc* tests performed as appropriate. The present study was conducted in accordance with the recommendations of the Research Ethics Committee of the UNED (Universidad Nacional de Educación a Distancia, Madrid). The UNED Institutional Review Board approved the study protocol. All participants provided written informed consent and were informed of their right to cease participation in the study at any time. The study was conducted in accordance with the Declaration of Helsinki (World Medical Association, 2013).

#### Cognitive evaluation: assessment tasks and procedures

The experimental tasks performed before and after training are described below. At the end of the last assessment session, participants answered questions regarding their study expectations and perceived improvement in the different tasks.

#### Attentional tasks

Distraction and alertness, two important functions of selective attention were assessed with a cross-modal oddball task. Effortful inhibitory control and automatic passive inhibition were measured with the Stroop task and the Negative Priming task, respectively, both included in a computerized task.

##### Cross-modal oddball attention task

As in our previous RCT (Ballesteros et al., 2014; Mayas et al., 2014), we assessed selective attention with a cross-modal oddball task. This would allow us to compare performance after non-action video game training when the control group was a passive control group (as in our previous study) with that of an active group (the present study). The task comprised three blocks of 384 trials each (24 practice trials and 360 test trials, as described below). In each trial, participants categorized a visual digit from 1 to 8 as odd or even by pressing one of two response keys (counterbalanced across participants). Each trial began with the presentation of a white fixation cross in the center of a black screen together with a 200 ms sound. The digit appeared in white in the center of the screen 100 ms after the sound's offset, and remained on the screen for 200 ms. A response window was displayed for 1,200 ms from the digit's onset. There were three sound conditions: A silent block and two blocks of trials containing two different sounds, the standard sound presented in 80% of the trials that was a 600 Hz sine wave tone of 200 ms, and a novel sound used in 20% of the trials taken from a list of 72 environmental sounds (e.g., hammer, drill, door, rain, etc.) used in Andrés et al. (2006). All sounds were normalized and presented binaurally through headphones at a constant volume. Participants were instructed to focus on the digit categorization task and ignore any sound, and to respond as fast and accurately as possible.

##### Stroop-negative priming (NP) task

Cognitive processes that involve top-down control mechanisms decline with aging, but more automatic processes do not (Ballesteros and Reales, 2004). The Stroop interference effect reflects the extra time needed to resolve the conflict generated by an automatically processed irrelevant dimension. NP and the standard Stroop effect were evaluated within the same task. In the standard NP procedure, participants are presented with pairs of prime and probe displays containing two stimuli, the to-be-responded target and the to-be-ignored distractor. In the critical trials, participants have to respond to a target that served as a distractor in the previous prime display (the ignored repetition condition). Reaction times (RTs) to targets in the ignored repetition condition are slower than in the control condition, in which the distractor in the prime display is not repeated as the target in the probe display (Tipper and Cranston, 1985; Tipper, 2001; Andrés et al., 2008). The aim was to investigate whether training older adults improved controlled, effortful inhibition (measured by Stroop interference) to a greater extent than automatic passive inhibition (NP).

The stimuli were three basic color words (“red,” “green,” and “blue”) written in red, green, or blue, presented in the center of the computer screen. Participants responded by pressing the appropriate key of the computer keyboard. Each trial started with a black fixation cross, presented in the center of the screen on a white background. Stimuli were presented randomly and remained on the screen for 200 ms. Participants responded as quickly and accurately as possible by pressing a key according to the color of the stimulus word while ignoring its semantic meaning. Participants performed a block of 18 practice trials (with feedback) and four experimental blocks of 144 trials each. Responses for the Stroop analysis were coded as a function of the congruency between the color and the meaning of the stimulus.

#### Working memory (WM)

Visuospatial WM was assessed with a computerized version of the Corsi Blocks task. Maintaining and updating in verbal working memory was evaluated with the *n*-back task.

##### Corsi blocks task

Visuospatial working memory (Baddeley and Hitch, 1974) was assessed with a computerized version of the Corsi task (Milner, 1971), similar to the task used in our previous intervention studies (Ballesteros et al., 2014; Toril et al., 2016), with six levels of increasing difficulty (2, 3, 4, 5, 6, and 7 cube positions) and 12 trials per level. The first two trials in each difficulty level were used as practice trials and were not analyzed. The stimuli consisted of black squares that appeared one by one in the center of the computer screen inside a 3 × 3 matrix for 1,000 ms each, with a 500 ms inter-stimulus interval (ISI). The positions in each sequence were randomly selected for each participant, the only restriction being that two cubes did not appear in the same position within the same trial. In each trial, participants were asked to reproduce the sequence of cubes in the same order as in the presentation. Participants responded by marking the presentation order of the cubes on a separate response sheet. They started the next trial by pressing the space bar. The final score was the proportion of correct sequences reproduced at each difficulty level.

##### N-back memory task

As a measure of maintenance and updating of information in WM, participants performed a verbal *n*-back task (Wayne, 1958). This task has been widely used to assess WM in young (Baniqued et al., 2015; Kable et al., 2017) and older adult training studies (Basak et al., 2008; Dahlin et al., 2008; Redondo et al., 2016). We used an adapted version of the Robinson and Fuller (2004) computerized task. Participants viewed a sequence of centrally presented stimuli (letters) and indicated whether the last stimulus was identical to the one presented “*n*” trials back by pressing one key for “yes” and another for “no.” In this task we used three difficulty levels (1, 2, and 3 back). In the one-back level, participants had to remember the item presented just before the current item; in the two-back level, they had to remember the item presented two positions before; and in three-back level, three positions before. The stimuli in all blocks were 20 consonants (B, C, D, F, G, H, J, K, L, M, N, P, Q, R, S, T, V, W, Y, and Z). The letters appeared one by one in the center of the computer screen (font: Palatino Linotype, size: 30) for 500 ms, with an ISI of 3,000 ms for participants' responses. The task started with a practice session of 17 trials at each level, followed by the experimental session and feedback. Each level contained three blocks of 27 trials, giving a total of 81 trials per level. Each block of 27 trials consisted of 17 “non-targets” (“no” response) and 10 “targets” (“yes” response).

##### Motivation and engagement self-reports

Motivation and engagement across the training period were assessed at pretest, at training sessions 1, 8, and 16, and at post-test. Participants responded on a 10-point Likert scale to questions about their motivation (“*How motivated were you to achieve the highest score on the game? 1* = *not motivated at all; 10* = *extremely motivated*”) and engagement (“*How engaging was each game? 1* = *not engaging at all; 10* = *extremely engaging*”).

##### Post-experiment survey

At the very end of the post-assessment session, participants answered questions related to whether they felt that participation in the study changed how they performed daily life activities, memory, processing speed, emotions, attention, and visual acuity, using a 5-point Likert scale (1 very much; 5 not at all). They also provided information about their expectations of improvement after training on the four transfer tasks.

#### Overview of the training program

Training was conducted in small groups of 10–12 participants at the UNED Associated Center in Madrid. At the beginning of each training session, the experimenter handed out an iPad (Brigmton BTPC 1018OC) to each participant. Participants in the experimental and the active control groups completed 16 training sessions of ~40–50 min each over 10–12 weeks. According to the results of our meta-analytic study (Toril et al., 2014), short training regimes are better than long ones. We therefore used a training regime that was not too long to avoid loss of motivation.

##### Cognitive training intervention with non-action games

In each session, the trainees in the experimental group played 10 non-action video games selected from the commercial *Lumosity* (http://www.lumosity.com/) computerized training program. Some video games from this platform are based on traditional psychological tasks. The video games are quiet-pace, short (3–6 min), and were designed to be engaging. All participants played the same video games. The selected video games claim to train the following specific cognitive domains: working memory (20% of the games), attention (30%), response inhibition (10%), task switching (20%) and speed of processing (20%). Table 2 provides a short description of the games and their trained domains. These video games were: *Playing Koi, Highway Hazards, Speed Match, Tidal Treasures, Star Search, Color Mach, Lost in Migration, Pinball Recall, Ebb and Flow, and Disillusion*. A main feature of these video games is that they are adaptive meaning that as performance improved, difficulty increased progressively.

**Table 2:** Short description of the 10 non-action video games played by the experimental group (selected from *Lumosity*).

**Game name**	**Trained function**	**Description**
*Tidal Treasures*	Working memory	The player has to choose objects and memorize their choice.
*Pinball Recall*	Working memory	The player has to predict a ball's path.
*Playing Koi*	Divided attention	The task consists of feeding some fish and remembering those that have already been fed.
*Star Search*	Selective attention	The player has to choose the odd-one-out in a group of objects.
*Lost in Migration*	Selective attention	A flock of birds appears on the screen and the player has to swipe in the direction the middle bird is facing.
*Color Match*	Response inhibition	The player has to compare one word's meaning to another word's color.
*Disillusion*	Task switching	The player has to match tiles with different shapes, colors or symbols.
*Ebb and Flow*	Task switching	Leaves appear on the screen and the player has to swipe in the direction they are moving or pointing.
*Highway Hazards*	Information processing	The player races a car across the desert avoiding colliding with obstacles.
*Speed Match*	Speed, information processing	A card appears on the screen and the player must determine whether the card is the same as the previous one.

##### Active control condition

Participants in the active control condition practiced video games not designed to train specific cognitive domains but allows to trigger expectancy, contact with the trainer, motivation, and novelty. Participants played the same number of sessions for the same time than the cognitive trained group. Game difficulty was not adapted each session to the user ability. Participants in this group started each training session from the beginning of the video game. It is important to mention that both groups had the same contact with the trainer as all the training sessions were conducted on the present of the trainer and in small groups. Moreover, participants in both conditions received the same completion incentives. The active control group played *The Sims* and *SimCity Build* (Electronic Arts Inc.), simulation strategy games. The Sims was used as control in previous intervention studies conducted with young adults (e.g., Oei and Patterson, 2013; Blacker et al., 2014). These simulation games did not appear to have the same cognitive demands as the behavioral tasks. They have some memory demands as the player has to keep track of the goals to achieve but it is not necessary to do so as the goals are available in the menu (see Oei and Patterson, 2013). For example, The Sims player created and controlled characters that accomplished and performed a number of tasks similar to real-life activities (making friends, sleeping, have a bath, find a job, and so on). SimsCity Build is also a simulation strategy game in which the player performed the tasks corresponding to the Mayor of the city. The task is to create and expand the city. Players are not required to accomplish objectives in a pre-determined order. Table 3 presents a short summary of these games.

**Table 3:** Short description of the life simulation games played by the active control group (*Electronic Arts, Inc*.).

**GAME NAME**	**DESCRIPTION**
*SimCity Build It*	Life simulation game in which the player is the Mayor of a city that he or she must expand.
*The Sims*	Life simulation game in which the player creates characters (*The Sims*) that live in a virtual world that is similar to the real one. *The Sims* have to work, build their own homes, develop relationships, etc.

## Results

We first examined whether there were practice-related improvements on the trained games across the 16 training sessions. Next, we analyzed whether video-game training gains transferred to untrained tasks by comparing baseline (pre-test) to post-test performance in each group. We also considered whether perceived improvements in attention and working memory differed between groups. Levels of motivation and engagement throughout the training were assessed from the participants' answers to the self-report questions at pretest, at the 1st, 8th, and 16th training sessions, and at post-test. Groups did not differ in motivation [*t*_(53)_ = 0.35, *p* = 0.72], engagement [*t*_(53)_ = 0.24, *p* = 0.81], or expectation [*t*_(53)_ = −1.26, *p* = 0.72] at pretest.

### Video game performance and game experience across sessions

**Figure 2:**
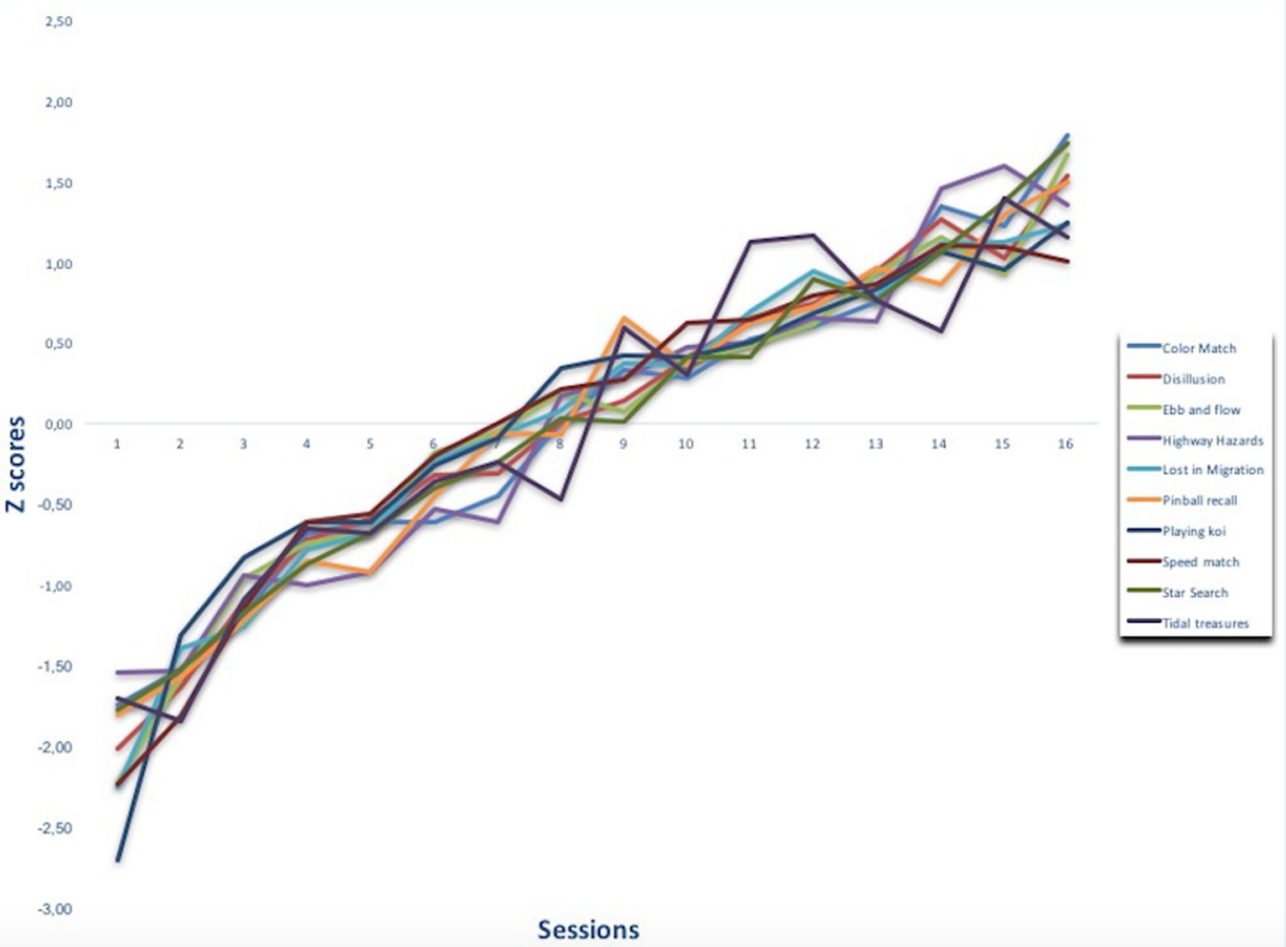
Average performance scores obtained in each video game across the training sessions in Z scores (mean 0; standard deviation 1).

The difficulty of the non-action video games was modified using an adaptive algorithm within and across the training sessions. The results showed that video game performance improved across sessions (see Figure 2). Comparisons between the first and last sessions were conducted on the performance Z-scores (Table 4). As expected, the results showed that training improved accuracy in all the video games.

**Table 4:** Performance (Z-Scores) of the 30 participants in the experimental group in the first and last training session on each of the 10 practiced non-action video games.

**Game**	***t*_(19)_**	***p-value***
Color match	1.99	0.02
Disillusion	16.2	0.00
Ebb and flow	15.28	0.00
Highway hazards	4.96	0.00
Lost in migration	19.64	0.00
Pinball recall	9.53	0.00
Playing koi	15.62	0.00
Speed match	15.50	0.00
Star search	21.77	0.00
Tidal treasures	3.77	0.00

#### Game experience across training sessions

Responses to questions on motivation (How motivated were you to achieve the highest score on the game?) and engagement (How engaging was each game?) were analyzed separately using mixed ANOVAs with group (experimental vs. active control) as a between-subjects factor, and training session (1, 8, and 16) as the within-subjects factor. Rating scores to these questions were averaged across all the games. The ANOVA conducted on motivation scores showed that neither the main effect of group [*F*_(1, 52)_ = 0.513, *MSE* = 8.350, *p* > 0.05] nor session [*F*_(2, 104)_ = 0.863, *MSE* = 1, 204, *p* > 0.05] were statistically significant. More importantly, the group by session interaction was marginally significant [$F_{\left(2,\;104\right)}=2.955,MSE=1.204,\;p=0.056,\;\eta_{Partial}^{2}=0.054$]. However, the simple effects analysis of this marginally significant interaction showed no effect whatsoever in the group variable across sessions, suggesting that the two groups were similarly motivated across sessions.

The ANOVA conducted on engagement showed that the two groups were similarly engaged [*F*_(1, 52)_ = 0.411, *MSE* = 7.07, *p* > 0.05], but engagement differed across sessions [$F_{\left(2,\;104\right)}=3.212,\;MSE=1.354,\;p>0.05,\eta_{Partial}^{2}=0.058$]. Pairwise comparisons showed that the scores at session 16 (post-test) were significantly lower (*x* = 7.588, *SE* = 0.241) than at session 8 (*x* = 7.971, *SE* = 0.213). The group by session interaction was also statistically significant [${F_{\left(2,\;104\right)}=3.376,\;MSE=1.354,\;p>0.05,\;\eta }_{Partial}^{2}=0.061$]. Simple effects analysis of this interaction did not show any effect of group on session, except the 16th, which showed a marginal *p-value* (*p* = 0.065), suggesting that the two groups were similarly engaged, although the experimental group showed a slight drop in engagement in the final part of the experiment.

#### Perceived improvement: post-assessment survey

Transfer expectations of video-game training on a series of different activities were assessed with a 5-point Likert scale (1 expectation of no change, and 5 expectation that training will have strong positive effects). A series of one-factor ANOVAs (group: experimental, active control) were performed to assess the perceived improvement of video-game training on daily life activities, memory, processing speed, current studies, emotions, attention and visual acuity. The results showed that groups did not differ in their expectations of improvement in daily life activities [*F*_(1, 52)_ = 0.91, *MSE* = 0.89, *p* > 0.05, $n_{P}^{2}$ = 0.02], memory [*F*_(1, 52)_ = 1.12, *MSE* = 1.27, *p* > 0.05, $n_{P}^{2}$ = 0.02], processing speed [*F*_(1, 52)_ = 2.48, *MSE* = 3.22, *p* > 0.05, $n_{P}^{2}$ = 0.05] or emotions [*F*_(1, 52)_ = 2.22, *MSE* = 2.50, *p* > 0.05, $n_{P}^{2}$ = 0.0]. The experimental group had higher expectations of improved attention [*F*_(1, 52)_ = 5.12, *MSE* = 6.23, *p* < 0.05, $n_{P}^{2}$ = 0.09] and visual acuity [*F*_(1, 52)_ = 5.69, *MSE* = 7.50, *p* < 0.05] after training than the control group.

To assess possible differences in expectation of improvement after training on the experimental tasks (Oddball task, Stroop-NP task, Corsi task, *N*-back task), participants indicated their expected improvement after video-game training on a scale of 1 to 5. ANOVAs revealed that groups did not differ in their expectations of improving their performance on the Oddball [*F*_(1, 53)_ = 0.91, *MSE* = 0.62, *p* > 0.05, $n_{P}^{2}$ = 0.02] and *N*-back tasks [*F*_(1, 53)_ = 0.52, *MSE* = 0.27, *p* > 0.05, $n_{P}^{2}$ = 0.01]. The ANOVAs revealed a significant group effect for the Stroop task [*F*_(1, 53)_ = 6.06, *MSE* = 3.69, *p* < 0.05, $n_{P}^{2}$ = 0.10] and the Corsi task [*F*_(1, 53)_ = 9.52, *MSE* = 4.80, *p* < 0.05, $n_{P}^{2}$ = 0.15], with higher ratings in the experimental than the active control group.

### Transfer of video-game training gains

**Figure 3:**
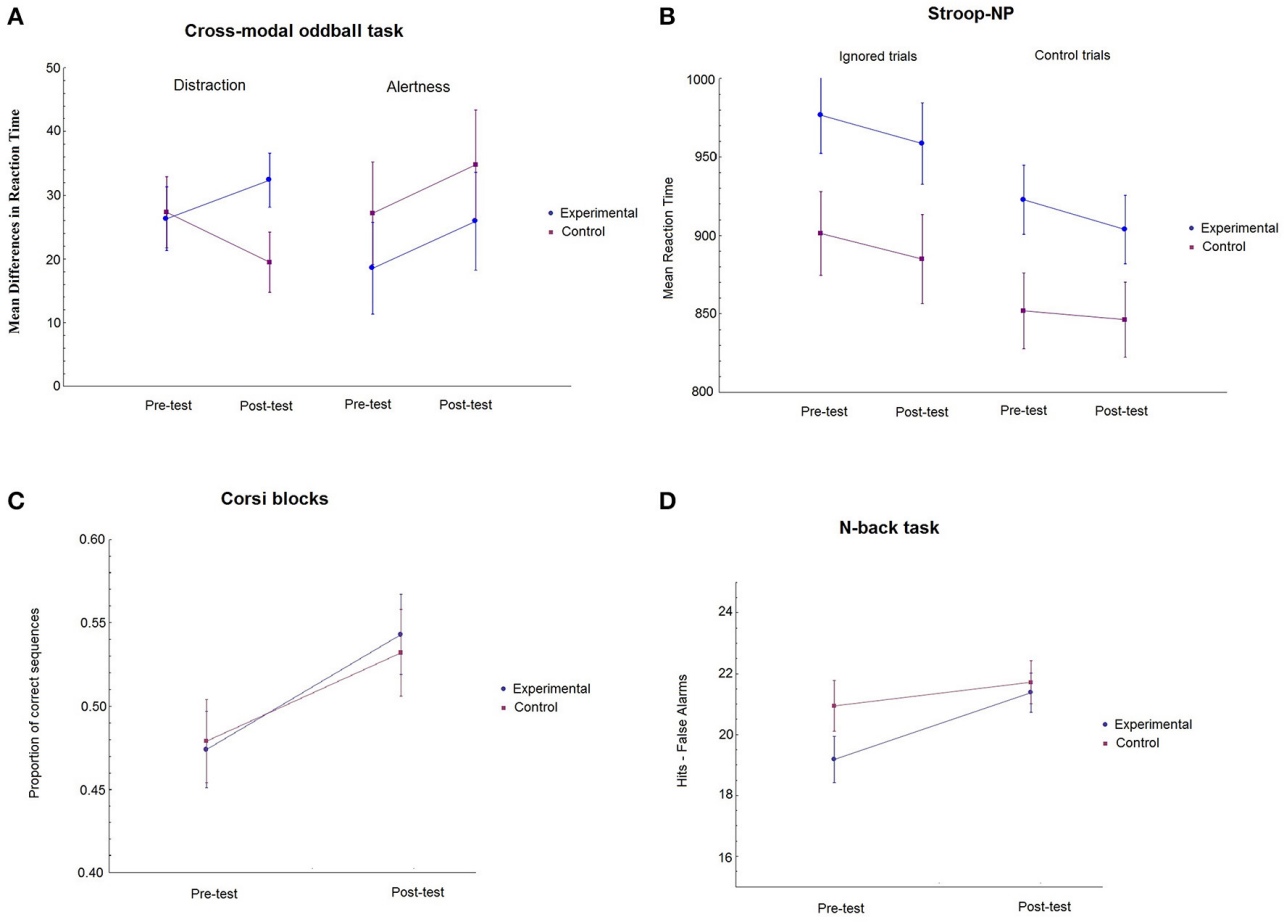
Mean performance of trained and active control groups at pre-test and post-test. **(A)** Mean differences between conditions for distraction (novel—standard) and alertness (silence—standard) in ms. **(B)** Mean RTs for ignored and control trials in the Stroop task. **(C)** Mean proportion of correct sequences in the Corsi blocks task. **(D)** Mean hits—false alarms rates obtained in the *n*-back task. Error bars represent plus minus 1 standard error.

The main results obtained in the transfer tasks at pre-training and post-training by the experimental and the active control groups are displayed in Table 5 and Figure 3.

**Table 5:** Pre and post-training performance on psychological measures for the experimental and active control groups.

		**Experimental group**		**Active control group**		
		**Pre**	**Post**	**Pre**	**Post**	**Pre-post effects (d) with Ci**
**Crossmodal oddball task**	Distraction (ms) +	26.32 (28.62)	32.37 (25.33)	27.33 (25.69)	19.4 (20.11)	−0.51 [−1.05, 0.03]
	Alertness (ms)	18.55 (23.87)	25.93 (31.38)	27.19 (52.8)	34.80 (52.56)	0.01 [−0.52, 0.54]
**Stroop NP-task**	Stroop effect (ms)	93.93 (42.38)	92.05 (39.41)	79.29 (32.31)	72.94 (29.29)	−0.11 [−0.64, 0.43]
	NP effect (ms)	53.91 (43.00)	54.74 (46.86)	49.26 (28.59)	38.66 (33.05)	−0.25 [−0.79, 0.28]
**Corsi blocks task** ++	2 Serial position	0.92 (0.11)	0.97 (0.04)	0.90 (0.21)	0.95 (0.05)	0.00 [−0.53, 0.53]
	3 Serial position	0.71 (0.16)	0.76 (0.14)	0.76 (0.16)	0.79 (0.14)	−0.13 [−0.66, 0.40]
	4 Serial position	0.60 (0.23)	0.67 (0.22)	0.66 (0.23)	0.71 (0.23)	−0.09 [−0.62, 0.45]
	5 Serial position	0.29 (0.23)	0.40 (0.27)	0.32 (0.26)	0.41 (0.24)	−0.08 [−0.61, 0.45]
	6 Serial position	0.16 (0.18)	0.27 (0.23)	0.15 (0.14)	0.20 (0.15)	−0.30 [−0.82, 0.25]
N-back task^*^	1-back (Hits–FA)	23.93 (4.42)	26.68 (2.03)	24.58 (3.47)	26.37 (2.53)	−0.27 [−0.80, 0.27]
	2-back (Hits–FA)	19.82 (5.73)	22.54 (3.18)	22.54 (3.18)	21.75 (5.84)	−0.73 [−1.28, −0.73]
	3-back (Hits–FA)	13.79 (5.43)	15.70 (6.26)	15.70 (6.26)	17.04 (4.85)	−0.10 [−0.63, 0.44]

Mean scores of the outcome measures with standard deviations in parentheses. Single asterisk (^*^) indicates tasks on which there was a trend for large improvements in the experimental group; the cross (+) indicates tasks on which the active control group showed significantly greater improvement than the experimental group and the double cross (++) indicates that both groups improved after training. Effect size (d) is the standardized mean difference for designs with two groups (experimental and control) in a pre-post test design. CI is the confidence interval of d.

#### Attentional functions

##### Cross-modal oddball task

The main dependent variables for this task were distraction and alertness. We did not apply an ANCOVA with the pre-test scores as covariates because the assumption of independence with the inter-subjects factor was not met. The 2 group (experimental vs. control) × 2 session (pre- vs. post-test) × 3 sound condition (silence, standard, novel) mixed ANOVA conducted on the mean of the RTs of the correct responses showed a main effect of sound condition [$F_{\left(2,\;104\right)}=\;33.79,\;MSe=1075.94,p\;<\;0.001,\;\eta_{p}^{2}=\;0.394$]. *Post-hoc* pairwise comparisons were all significant: standard sound condition was faster (622 ms) than the silence (645 ms) and novel (658 ms) conditions (*ps* < 0.001), while mean RT of the silence condition was faster than that of the novel condition (*p* = 0.03). The analysis also yielded a main effect of group *[*$F_{\left(1,\;52\right)}=\;5.70,\;p\;=\;0.021,\;MSe=26573.78,\;\eta_{p}^{2}=\;0.10$]. This result indicates that the active control group was faster (620 ms) than the experimental group (664 ms) overall. The main effect of session was also significant [$F_{\left(1,\;52\right)}=\;11.01,\;p\;=\;0.002,\;MSe\;=1087.59,\;\eta_{p}^{2}=\;0.175$]. RTs were faster at post-test (636 ms) than pre-test (647 ms). Finally, the session × sound interaction was statistically significant [$F_{\left(2,\;104\right)}=4.69,MSe=717.65,\;p\;=\;0.017,\;\eta_{p}^{2}=\;0.08$]. *Post-hoc* comparisons indicated that RTs were faster at post-test only in the standard (617 and 627 ms for pre- and post-test, respectively) and novel (646 and 670 ms for pre- and post-test, respectively) conditions (*p* = 0.05 and *p* < 0.001, respectively). Additional analyses were conducted on distraction and alertness. We computed the distraction effect as the difference between the RT of the novel trials and the RT of the standard trials. A 2 group × 2 session ANOVA was performed on the *distraction* effect. Only the two-way group × session interaction was significant [$F_{\left(1,\;52\right)}=\;5.7,\;MSe=\;222.53,\;p\;=\;0.020,\;\eta_{p}^{2}=\;0.10$], indicating that the active control group improved at post-test (27.33 and 19.49 for pre- and post-test, respectively) but the experimental group did not (26.32 and 32.37 for pre- and post-test, respectively). We computed the *alertness* effect as the difference between the RT of the silence trials and the RT of the standard trials. The ANOVA conducted on *alertness* did not show any significant effect (all *ps* > 0.05). Figure 3A represents the difference in RTs between conditions for *distraction* and *alertness*.

##### Stroop—NP task

*Stroop results* Responses for the Stroop analysis were coded as a function of the congruency between the color and the meaning of the color word. Congruent trials were those in which the color of the word coincided with the color in which it was presented. Incongruent trials were those in which the color word did not coincide with the color in which it was displayed. Trials were also coded according to the congruency of the word in the previous trial (N-1) with the color in the current trial in order to compute the NP effect. Thirty participants in the experimental group and 25 participants in the control group were included in this analysis.

To analyze the results of the Stroop task, a 2 group × 2 session × 2 congruency (congruent and incongruent) mixed ANOVA was conducted on the mean RTs for correct trials as the dependent variable. The analysis showed a highly significant main effect of congruency (classic Stroop effect) [$F_{\left(1,\;53\right)}=\;345.62;\;MSe=109.86,\;p<0.001,\;{\;\eta }_{p}^{2}=\;0.867$]. Responses to the incongruent stimuli were slower (884 ms) than to the congruent ones (801 ms; *p* < 0.001). The main effect of group was significant [$F_{\left(1,\;53\right)}=\;4.70,\;MSe\;=\;40729.29,\;p<0.05,\;\eta_{p}^{2}=\;0.081$], showing that the control group was faster (813 ms) than the experimental group (872 ms). The significant group × congruency interaction qualifies the main effect of group [$F_{\left(1,\;53\right)}=\;4.53,\;MSe=1098.86,p<0.05,\;\eta_{p}^{2}\;=\;0.08$]. *Post-hoc* comparisons revealed that the active control group was faster than the experimental group (850 and 919 ms for the control and the experimental group, respectively), but only for incongruent stimuli (*p* < 0.02). Finally, we computed the Stroop effect as the difference between Incongruent RT—Congruent RT for each participant. A 2 group × 2 session mixed ANOVA showed that only the main effect of group was significant [*F*_(1, 53)_ = 4.53, *MSe* = 2197.72, *p* < 0.05, $n_{P}^{2}$ = 0.08]. No other main effect or interaction was statistically significant. See Figure 3B.

*NP results* Responses for NP analyses were coded as a function of the relationship between the color of the current target word and the color denoted by the word in the previous trial (distractor). Different types of trials were coded: (a) ignored repetition trials were those in which the word in the preceding trial denoted the color of the word color of the current stimulus; and (b) control trials were those in which both the target (color) and the distractor (word) in the current trial were different from the target and distractor in the previous trial. The ignored repetition condition and the control condition were always an incongruent trial preceded by an incongruent trial.

A 2 group × 2 session × 2 repetition (ignored repetition, control condition) mixed ANOVA was performed on the mean RTs for correct trials. The NP effect was computed as the difference between ignored repetition and control conditions. The analysis showed a significant main effect of group [$F_{\left(1,\;53\right)}=\;4.45,\;MSe\;=\;58822.53,\;p<0.05,\;\eta_{p}^{2}=\;0.07$], indicating that the experimental group was significantly slower (941 ms) than the control group (871 ms) in all conditions. The main effect of repetition was also significant [???, showing that ignored repetition trials were slower than control trials for both groups (for the experimental group: 968 and 913 ms for ignored repetition and control trials, respectively; for the control group: 893 and 849 ms for ignored repetition and control trials, respectively). The size of the NP effect was 55 ms for the experimental group and 44 ms for the control group. Finally, neither the main effect of session nor any interactions were significant (all *p*s > 0.05).

#### Effects of training on spatial working memory

##### Corsi blocks

We conducted a 2 group × 6 level ANCOVA with the pre-test scores as covariates conducted on the proportion of correct sequences obtained at each difficulty level. The assumption of no relationship between the inter-subjects factor (group) and the covariates was met (all *p*_*s*_ > 0.05) as well as the equality of slopes (*all p*_*s*_ > 0.05). The results showed a main effect of level [*F*_(5, 235)_ = 13.706, *MSe* = 0.022, *p* < 0.001, $\eta_{p}^{2}=$ 0.382]. The mean correct proportions were 0.97, 0.78, 0.69, 0.41, 0.24, and 0.13 for levels 2, 3, 4, 5, and 6, respectively. *Post-hoc* pairwise comparisons showed that all levels differed significantly from each other (all *p*s < 0.05). Neither other main effects nor any interaction reached statistical significance. However, as the ANCOVA did not include session as a factor, we also performed an ANOVA to assess specifically the effect of session. This analysis showed that session was highly significant [*F*_(1, 53)_ = 22.57, *MSe* = 0.027, *p* < 0.001, $\eta_{p}^{2}=$ 0.299]. The mean correct proportion at pre-test was 0.48 while at post-test the mean was 0.54. The interaction session × group was not significant, suggesting that both groups benefited equally after training (see Figure 3C).

##### N-back task

One participant in the experimental group and one in the control group were excluded from the analysis due to the large number of no responses (more than 50%). Thus, data from 29 participants in the experimental group and 24 participants in the control group were included in this analysis. We did not perform an ANCOVA with the pre-test scores as covariate because the assumption of no relationship between the inter-subjects group factor and the covariates was not met. The 2 group × 2 session × 3 level mixed ANOVA conducted on the means of Hits minus False Alarms yielded a significant main effect of Level [$F_{\left(2,\;102\right)}=188,68,\;MSe\;=\;13,21,\;p\;<0.001,\;\eta_{p}^{2}=\;0.79$] with means of 25.39, 21.33, and 15.69 for 1-back, 2-back, and 3-back levels, respectively. A main effect of session also reached significance [$F_{\left(1,\;51\right)}=13.91,\;MSe\;=\;12.51,\;p<\;0.001,\;\eta_{p}^{2}=\;0.21$]. The Hits-FA mean was 20.06 at pre-test and 21.55 at post-test. The session × level interaction was marginally significant [$F_{\left(2,\;102\right)}=\;3.06,\;MSe\;=\;9.48,\;p\;=\;0.051,\eta_{p}^{2}\;=0.06$7], showing that only 1-back and 3-back levels improved between pre- and post-test. Finally, the group × session interaction was also marginally significant [$F_{\left(1,\;51\right)}=3,162,\;MSe\;=12.518,\;p=\;0.08,\eta_{p}^{2}\;=\;0.06$], suggesting that the experimental group improved marginally at post-test compared to the control group (19.18 and 20.94 at pre- and post-test for the experimental group; 21.37 and 21.72 at pre- and post-test for the control group). Figure 3D shows this marginal interaction.

## Discussion

The present study yielded three main results: (1) Unsurprisingly, participants improved significantly in the video games across the training sessions; (2) the experimental group did not show greater improvements in measures of selective attention and working memory compared to the control group; and (3) a marginal training effect was observed for the *N*-back task, but not for the Stroop task in the experimental group while both groups improved similarly in the Corsi Blocks task. On the basis of these results, one can conclude that training with non-action video games provide modest benefits for untrained tasks (near transfer) that were not directly trained but were under the umbrella of the executive function. Moreover, the effect was not specific for that kind of training, since a similar effect was observed in the group trained with simulation strategy video games.

Older adult participants in the non-action adaptive video games improved on all the practiced games across the training sessions. This is in line with previous findings reported in a large number of intervention studies (e.g., Ackerman et al., 2010; Reddick et al., 2013; Ballesteros et al., 2014; Baniqued et al., 2014; Toril et al., 2016).

### Transfer effects

Motivated by results suggesting that training older adults with non-action video games improve aspects of cognition when compare performance with that of a passive control group (Ballesteros et al., 2014; Toril et al., 2016), and by others showing that executive control functions could improve with adaptive computerized training (Morrison and Chain, 2011; Nouchi et al., 2013; Hardy et al., 2015), we hypothesized that older adults trained with adaptive cognitive video games would show greater improvements in measures of executive function than an active control group trained with video games not designed specifically for cognitive training. The experimental group trained with the commercial adaptive games showed a trend to improve in the *N*-back task after training and a similar degree of improvement on the Corsi task than the active control group. The opposite occurred in the Oddball task in which only the active control group showed less distraction after training. Based on these results, one might conclude that training with non-action games provide modest benefits for untrained tasks compared with an active control group trained with non-adaptive simulation strategy video games.

A recent randomized controlled trial conducted with 64 healthy young adults, trained with the same commercial web-based cognitive training video games and 64 trained with web-based video games that do not target executive functions or adapt the difficulty during training (active control group) found that performance over time in the cognitive training group improved across sessions in the trained video games. However, both training conditions improved similarly in the cognitive assessment battery that included tests of attention, working memory (visual/spatial *n*-back), response inhibition, interference control (Stroop test), and cognitive flexibility, not directly trained but in the domain of executive function targeted by the training regimen (Kable et al., 2017).

It is central to consider the importance of the control group, as most previous intervention studies did not include an active control condition as similar as possible to the experimental condition (Lampit et al., 2014; Toril et al., 2014) and with similar levels of motivation, social contact, and engagement (Blacker et al., 2014; Motter et al., 2016). In the current RCT, we tried to equate task factors that might contribute to differential improvements. First, the number of older participants was almost double that of our previous intervention studies. They were randomly assigned to a group trained with video games from *Lumosity* or to an active control group playing *The Sims* and *SimCity* (two simulation strategy games) for the same number of sessions. The inclusion of an active control group is considered critical for inferences about the specific potential effects of the intervention (Dougherty et al., 2016; Motter et al., 2016; Simons et al., 2016). We selected an active control condition as similar as possible to the training condition in that the control group also played non-action games. In several recent intervention studies conducted with young adults to investigate whether training with action video games enhances aspects of cognition, including visual WM (Blacker et al., 2014), cognitive flexibility (Glass et al., 2013), plasticity in the visual system (Li et al., 2011) or several aspects of perception and cognition (Oei and Patterson, 2013), the active control participants also played non-action strategy video games.

Boot et al. (2011; see also Dougherty et al., 2016; Simons et al., 2016) warned that the use of a control group *per se* does not preclude the possibility of differential placebo effects contaminating the results of the trained group. The argument is that the experimental group might have higher expectations of their performance on the transfer tasks compared to the active control group. Recently, Blacker et al. (2014) collected measures of expectations in a group trained on action games and in an active control group trained with *The Sims*. Baniqued et al. (2014) used casual video games to train young adults. The intervention included an active *control* group that played several games not related to WM and reasoning, and responded to feedback questions about engagement, motivation, enjoyment, and perceived effort.

In order to match the expectations of our experimental group (trained with non-action games) with those of the active control group, we evaluated both groups' expectations for improvement on each outcome measure. Results showed that groups did not differ in their expectations of improvement in the attentional Oddball task and the verbal WM *N*-back task. The experimental group had higher expectations of improvement than the active control group in the response inhibition Stroop task and the visuospatial WM Corsi blocks task. The expectations and outcomes of the two groups were not aligned, so it is unlikely that the results were driven by a placebo effect.

There are discrepancies between the findings of the present training study and those of Toril et al.'s (2016) intervention study, which reported significant improvement by the experimental group after training with non-action video games in two computerized spatial WM tasks, the Corsi blocks and the Jigsaw puzzle task, and no change in a passive control group. In the present study, both groups improved their performance on the Corsi blocks after training. This suggests that playing strategy games also enhances visuospatial WM. This specific difference could be due to the fact that the games played by the active control group involve not only managing the characters' lives or a city but also traveling visually around the city to identify resources and opportunities. This visual navigation may be partly responsible for the results obtained in the visuospatial WM task. The discrepancy between the results of the two studies might thus be due to the type of control group, either passive (Toril et al., 2016) or active (the present study).

As indicated above, participants were randomly assigned to either the experimental group or the active control group before performing the cognitive tasks in the laboratory (pre-testing). However, the active control group was faster than the group trained with non-action cognitive video games in those cognitive tasks in which the dependent variable was response time (Oddball and Stroop-NP tasks). In the working memory tasks (Corsi Blocks and *N*-back tasks) in which the dependent variable was accuracy, experimental and control groups did not differ.

## Limitations

A number of limitations of the present study need to be acknowledged. First, although the number of participants in the present study was larger than many previously published training studies, it is always desirable to include a large number of participants per condition to increase power. Null effects may reflect the lack of power and variability within the groups. For example, Melby-Lervåg et al. (2016) advised that studies with small sample sizes (<20 participants per condition) and passive (untreated) control groups produce a bias toward significant (although low-powered) results (see also Maraver et al., 2016). In the current study, there were more than 20 participants per group and the active control group was also trained with video games. Secondly, it is possible that the 16 training sessions were insufficient to show transfer, and that a longer or denser (more hours per week) training regime could have yielded greater enhancements. However, as mentioned in the Introduction, recent meta-analyses (Lampit et al., 2014; Toril et al., 2014) showed that shorter training regimes were better than longer ones that can lead to loss of motivation. For that reason, we decided to have only 16 training sessions in the current study. Thirdly, as mentioned above, in two previous studies, we included a passive control group. In the present study we did not include a passive control group to control for unspecific repetition effects. Instead, in this RCT, we included an active control group and almost double the number of participants compared to our previous studies (Ballesteros et al., 2014; Toril et al., 2016). The inclusion of a passive control group would not have determined whether the improvements observed were due to the specific video games used in the training regimes, to the use of iPads, or simply to social interaction with the trainer and the other participants during the training sessions (see Ballesteros et al., 2015b; Schmicker et al., 2016). Even if a significant group by session interaction would be found, the result could be due to different factors as mention in the section Introduction. Finally, the participants in the present study were older adults without cognitive impairment. It could be the case that these elders were performing at a high level and training with the video games would not produce greater benefits (Toril et al., 2014). In fact, Kable et al. (2017) in their RCT with young adults included both, an active control group and a passive control group assessed at pretest and post-test without any training. The results showed that the improvement observed in the passive group was comparable to that of the group trained with adaptive commercial cognitive games and the active control group (Kable et al., 2017).

## Conclusion

In sum, further research is needed to ascertain whether computerized cognitive training improves executive functions, specifically selective attention and working memory, as well as everyday functioning in healthy older adults. While high levels of mental activity have been associated with both better cognitive performance and reduced risk of dementia (Valenzuela and Sachdev, 2006), in view of the modest benefits for untrained working memory and attentional functions of non-action video game training is vital to explore more deeply whether video games or other types of computerized cognitive training can improve executive functions in older adults (Foroughi et al., 2016; Motter et al., 2016; Simons et al., 2016) and whether there are stable relations between training with video games and cognitive abilities in general (McCabe et al., 2016). Special attention deserves multi-domain interventions that combine cognitive training with physical activity embedded in a social environment for supporting cognition and independent living of an increasing older adults population (Ballesteros et al., 2015a).

## Ethics statement

This clinical trial is registered on the ClinicalTrials.gov database (Clinicaltrials.gov ID: NCT02796508). The UNED's Ethical Review Board approved the trial. All the participants gave their written informed consent before the study started and were informed of their right to terminate participation at any time. The work described has not been published previously.

## Author contributions

Conceptualization and study design: SB and JM; Programming experimental tasks; JM and AP; ER enrolled the participants, conducted the training sessions and collected the data with some help from other members of the group; Data analysis: JM, PT, AP, and JMR; Questionnaires: ER and JMR; Interpretation: All the authors; Manuscript preparation: SB with some support from JM, JMR, ER, AP, and PT; Final approval: All the authors.

### Conflict of interest statement

The authors declare that the research was conducted in the absence of any commercial or financial relationships that could be construed as a potential conflict of interest.
